# Viral architects of the deep biosphere: reshaping the framework of sedimentary biogeochemistry

**DOI:** 10.1128/aem.00486-26

**Published:** 2026-04-30

**Authors:** Chong Wang, Chaomin Sun

**Affiliations:** 1 Laboratory of Experimental Marine Biology & Center of Deep Sea Research, Institute of Oceanology Chinese Academy of Sciences 53014 , Qingdao, China; 2 Shandong Province Key Laboratory of Marine Biodiversity and Bio-resource Sustainable Utilization, Institute of Oceanology Chinese Academy of Sciences 53014 , Qingdao, China; 3 Laboratory for Marine Biology and Biotechnology, Qingdao National Laboratory for Marine Science and Technology 474988 , Qingdao, China; 4 College of Earth Science, University of Chinese Academy of Sciences 74519, Beijing, China; Michigan State University, East Lansing, Michigan, USA

**Keywords:** viral ecology, marine sediments, biogeochemistry

## Abstract

A recent minireview by J. R. A. Williams and J. F. Biddle (Appl Environ Microbiol, 92:e00275-25, 2026, https://doi.org/10.1128/aem.00275-25) substantially reframes our understanding of sedimentary viruses. For decades, viruses in marine sediments have been viewed primarily as agents of mortality, their roles largely confined to the canonical “viral shunt” paradigm developed for pelagic systems. The authors expand this perspective, positioning viruses as active participants in benthic biogeochemistry—contributing to nutrient cycling, modulating microbial diversity, and influencing organic matter processing and carbon sequestration. This conceptual shift highlights sedimentary viruses as an integral and, until now, underappreciated component of global element cycles.

## COMMENTARY

## THE BENTHIC BLACK BOX

The ocean floor is not merely a graveyard for sinking detritus; it is a dynamic crucible where the planet’s long-term carbon budget is fundamentally forged. For decades, benthic carbon cycling has been primarily understood as driven by the metabolic activities of bacteria and archaea. Yet, embedded within these microbial communities—the very engine of deep-sea element cycling—resides a highly abundant and largely enigmatic biological component: the sedimentary viruses (1, 2). Marine virology has historically prioritized the water column, where viruses shape microbial communities and mediate carbon flux primarily through cell lysis and the recycling of dissolved organic matter (3, 4). Sediments, despite harboring Earth’s largest organic carbon repository and viral abundances reaching 10^10^ per gram, have remained comparatively unexplored (1). Williams’ and Biddle’s minireview (5) effectively dismantles this historical bias: viruses in marine sediments are not passive environmental residues but active, intrinsic participants in biogeochemical cycling, exerting influence through mechanisms that extend far beyond simple host mortality.

## ENDEMISM AND THE HIDDEN VIROME

The authors systematically dismantle the notion that sedimentary viruses are merely allochthonous inputs from the overlying water column. Metagenomic and experimental evidence reveals remarkable viral endemism in benthic environments (6–8). The composition of key viral guilds, such as Siphophages, differs by nearly 30% between sediments and the water column (6). Furthermore, unique viral morphotypes persist across disparate sedimentary habitats—from the chemically reducing environment of cold seeps to the high-temperature flux of hydrothermal vents (7, 8). This intrinsic endemism carries profound mechanistic implications: if sedimentary viruses replicate *in situ* and co-evolve with their hosts over geological timescales, their ecological and biogeochemical roles must be understood as locally adapted control systems, rather than generalized external threats. Metagenomic analyses further indicate that viral diversity is significantly higher in marine sediments than in the water column (6, 9), with current estimates suggesting a vast reservoir of unexplored viral taxa. Recent studies have begun to uncover how deep-sea viruses interact with specific hosts, such as the induction of chronic bacteriophages in *Lentisphaerota* strains by polysaccharides, revealing novel virus-host dynamics in the deep biosphere (10). Further work demonstrates that this substrate-specific viral induction extends to other key metabolisms: nitrogen sources trigger chronic phage release from deep-sea *Planctomycetota* (11), while nucleic acids induce a chronic phage in the wall-less bacterium *Hujiaoplasma nucleasis* (12). These chronic bacteriophages may assist host metabolism through their auxiliary metabolic genes, facilitating adaptation to energy-limited deep-sea environments. This hidden virome represents a genetic and functional reservoir whose influence on benthic biogeochemistry we are only beginning to appreciate.

## THE VIRAL SHUNT AS A GEOCHEMICAL LEVER

Perhaps most striking is the authors’ synthesis of how viral infection actively reorganizes benthic microbial ecology. Through the “killing the winner” dynamic, viral lysis prevents competitive exclusion by dominant taxa, thereby maintaining the exceptionally high microbial diversity that defines marine sediments (13, 14). This sustained biodiversity, in turn, underpins the metabolic versatility required to degrade complex, recalcitrant organic matter (15). While the concept of the “viral shunt”—the rapid recycling of organic matter through cell lysis—is well established in the water column (3, 4), applying this framework to the sediment necessitates a paradigm shift. In the deep sea, where energy is chronically limiting, viral lysis functions not merely as a destructive force, but as a sophisticated geochemical lever. By converting recalcitrant cellular biomass into bioavailable dissolved organic carbon and labile nutrients, viruses effectively “prime” the microbial community for heightened metabolic output (16, 17). This process is far from a zero-sum game of cellular attrition; it is a nuanced regulatory feedback loop. The viral shunt delivers an estimated 0.37–0.63 Gt of carbon annually to sediments (18), actively supplementing nutrient-depleted benthic communities with organic nitrogen and phosphorus that sustain microbial activity at depths where energy fluxes would otherwise prove limiting (17). Crucially, the organic material liberated from viral lysis provides approximately 2%–11% of the organic nitrogen and phosphorus essential for sedimentary processes (17), directly fueling enhanced microbial activity (16–19). This raises a profound, previously unappreciated possibility: if viral lysis provides the limiting cofactors required to boost bacterial activity, then the viral shunt may paradoxically accelerate the transformation of labile carbon into recalcitrant compounds (16, 18, 20). We are thus compelled to confront a critical question: do viruses act as the ultimate protectors of the global carbon sink, or are they the hidden catalysts of its release? Reconciling these dual roles is now a primary imperative for understanding the stability of Earth’s carbon reservoirs.

## BEYOND LYSIS: HORIZONTAL GENE TRANSFER AND METABOLIC ENGINEERING

The authors also highlight horizontal gene transfer (HGT) as a potent biogeochemical force. Lysogenic viruses, which predominate in sediments (6, 21), act as conduits for auxiliary metabolic genes (AMGs) that dynamically expand host metabolic capacities. Genes governing sulfate reduction, polysaccharide degradation, and phosphorus metabolism are frequently encoded in viral genomes, effectively functioning as mobile genetic modules that disseminate key biogeochemical functions throughout the microbial community (7, 21, 22). In hydrothermal vent sediments, where sulfur compounds abound, this gene transfer assumes particular significance, potentially driving the specialized metabolic networks that sustain these extreme ecosystems (21). The role of viruses in host metabolism transcends simple gene transfer; recent work demonstrates that deep-sea viruses can directly influence host metabolic pathways involved in the degradation of complex organic matter, effectively acting as metabolic engineers that reshape the functional potential of benthic microbial communities (23). The release of exogenous DNA via lysis provides a critical source of nitrogen and phosphorus (17) and can be integrated into microbial genomes (24), allowing traits such as pollutant degradation to proliferate within the community. In sediments of the Bohai, Yellow, and East China Seas, AMGs governing phosphate metabolism constitute a significant proportion of the viral community (22). This input of AMGs enables host microbes to rapidly adapt to environmental stresses (7). Crucially, this evolutionary resilience is rarely accounted for in current geochemical models. As the ocean environment undergoes rapid transitions—through warming, acidification, or pollution—the viral reservoir may facilitate microbial adaptation at a rate that exceeds that of traditional horizontal gene transfer alone. Thus, the viral community acts as an adaptive buffer against environmental change, yet we currently lack the predictive capacity to anticipate how this buffer will modulate ecosystem stability under future climate scenarios.

## DIRECT EFFECTS ON SEDIMENTARY GEOCHEMISTRY

Beyond lysing cells, viruses may function as essential reservoirs for dissolved organic phosphorus, modulating nutrient availability independently of host dynamics (25). The proportionality between viral abundance and organic matter concentration with sediment depth suggests that viruses actively maintain the organic carbon pool, effectively counteracting the expected depletion driven by diagenetic consumption (14, 16). This observation directly challenges conventional models of carbon burial and necessitates a comprehensive reconsideration of how organic matter persists within deep biosphere sediments. Viral lysis also releases carbon from the microbial community, with an estimated 0.37–0.63 Gt C yr⁻¹ released from the ocean into the atmosphere as a result of viral interactions (20). This value is remarkably comparable to the total carbon exported to sediments (18), presenting compelling evidence that viruses exert an influence on carbon release that is equal in magnitude to their role in sedimentation. This geochemical duality—simultaneously promoting burial and driving release—positions viral activity as a critical, yet overlooked, pivot point in the global carbon cycle.

## ENVIRONMENTAL VARIATIONS AND UNKNOWNS

The authors thoughtfully navigate the complex environmental mosaic across distinct sedimentary ecosystems. Shallow, coastal sediments exhibit a coupling of viral–microbial interactions with temperature, where elevated thermal conditions correlate with intensified activity (19)—a thermodynamic constraint that largely abates in the high-pressure, thermally stable deep-sea environment (14). Intriguingly, while pressure is a defining selective force in the deep benthos, it appears to exert negligible direct influence on viral-mediated microbial turnover (18). Despite these ecological divergences, the fundamental geochemical impacts of viral activity remain remarkably consistent across diverse regimes. However, as Williams and Biddle underscore, this synthesis also exposes critical knowledge gaps that define the next decade of discovery: freshwater sediments remain severely understudied (26), and marsh ecosystems—now recognized as pivotal “blue carbon” hotspots—lack even rudimentary viral characterization. Furthermore, the “viral shuttle,” wherein infection-induced particle aggregation accelerates the vertical flux of organic matter, may deliver carbon to the benthos far more efficiently than currently quantified (27, 28). Bridging these spatial and ecological divides is essential to transforming our fragmented observations into a unified theory of benthic viral influence.

## TOWARDS A SYSTEMS BIOLOGY OF THE DEEP BENTHOS

Why has the viral role in sedimentary geochemistry remained so elusive? The technical hurdles are, admittedly, monumental. Unlike the pelagic ocean, where viruses are predominantly free-floating, sediment viruses are inextricably bound to mineral matrices and flocculent layers, complicating isolation and quantification (1). Moreover, the inherent heterogeneity of sedimentary environments precludes the existence of a “universal model,” demanding instead a spatially explicit understanding of viral impact. Anthropogenic stressors further complicate this landscape; the loss of microbial biodiversity under fishing grounds and community shifts induced by oil contamination suggest a profound, albeit poorly understood, vulnerability in benthic biodegradation capacity.

Given the escalating imperative of carbon sequestration in climate mitigation strategies, future research must shift from descriptive census-taking to mechanistic integration. We must address how viruses modulate the potential for long-term sequestration versus the microbial shaping of burial-resistant organic matter. The future of this field lies in integrating multi-omic architectures with precise, *in situ* geochemical flux measurements. We advocate for a “systems biology” approach to sediment research—one where viral abundance, auxiliary metabolic gene diversity, and carbon-degradation kinetics are measured in tandem. Only through such a holistic, multidisciplinary framework can we reconcile the paradox of viral activity: that these entities, while individually destructive, are collectively essential for the stability and resilience of the global carbon sink.

## CONCLUSION

As atmospheric CO_2_ concentrations continue to escalate and marine sediments gain prominence in natural climate mitigation strategies, the model advanced by Williams and Biddle carries profound and urgent relevance. If viruses enhance carbon burial through the mechanisms they synthesize, then sedimentary carbon sequestration represents not merely a physicochemical process but a biologically mediated phenomenon contingent upon intact viral–microbial communities. Conversely, if viral lysis releases significant carbon back to the water column and atmosphere—comparable in magnitude to the amount buried—then accurate carbon accounting requires integrating viral dynamics into predictive models.

Marine virology stands where oceanography stood a century ago: transitioning from descriptive natural history to mechanistic understanding with predictive power. Williams and Biddle provide both a roadmap and an impetus. By placing viral processes squarely in the overlapping realms of evolutionary biology, microbial ecology, and biogeochemistry, they have brought intellectual refreshment and excitement to thinking about one of the most profound—and until now, most hidden—components of Earth’s biosphere. Ultimately, further sustained efforts in elucidating the nature of virus–bacteria interactions and their precise effect on geochemical flux will undoubtedly enhance our understanding of fundamental geochemical cycles while also offering crucial context regarding how anthropogenic pressures might reshape these foundational processes in the context of global climate stabilization.
